# Are TKIs favourable for the elderly with non-small-cell lung cancer?

**DOI:** 10.18632/oncotarget.9389

**Published:** 2016-05-17

**Authors:** Sabrina Rossi, Ettore D'Argento, Giovanni Schinzari, Vincenzo Dadduzio, Vincenzo Di Noia, Alessandra Cassano, Carlo Barone

**Affiliations:** ^1^ Department of Medical Oncology, Catholic University of Sacred Heart, Largo A. Gemelli, Rome, Italy

**Keywords:** gefitinib, afatinib, elderly, concomitant medications, efficacy, Gerotarget

## Abstract

**Background:**

Epidermal Growth Factor Receptor (EGFR) tyrosine-kinase inhibitors (TKIs) have changed treatment strategies for patients with advanced non-small-cell lung cancer (NSCLC) harbouring mutations in EGFR gene. This retrospective analysis assessed efficacy and safety of TKIs in elderly compared to younger patients.

**Patients and methods:**

49 patients with advanced NSCLC and mutations in exon 19 or 21 receiving a first-line therapy with TKIs were included and divided into patients aged <70 years and patients aged ≥ 70 years. Primary endpoints were progression free survival (PFS), response rate (RR) and clinical benefit in terms of quality of life; secondary endpoint was overall survival (OS).

**Results:**

Median PFS was significantly longer in elderly in comparison to younger patients (12.6 and 5.6 months, respectively; *p*=.008). RR was 64% in younger patients and 75% in elderly population. Eighteen out of 20(90%) elderly patients treated with gefitinib experienced symptoms relief and upgrading of performance status. No difference in terms of OS was found (*p*= .34).

**Conclusion:**

TKIs seem more effective in elderly than in younger patients affected by NSCLC with an EGFR gene mutation. We hypothesize that the main difference between the two populations is the number of medications related to concomitant comorbidities that cause an increased plasma level of TKIs.

## INTRODUCTION

Lung cancer is the leading cause of cancer-related death worldwide; about 75-80% of lung cancers have a non-small-cell histology (NSCLC). [1] Due to prolongation of life expectancy, most of cases are diagnosed in patients over the age of 65 and the median age at diagnosis is 70 years. [2]. In elderly patients, comorbidities, polypharmacy, impaired liver or kidney function are common and influence treatment decisions. Many studies evaluating efficacy and safety of chemotherapy in the elderly suggest that performance status and comorbidities have a greater prognostic importance than age itself. [3, 4].

In the last decade the discover of new molecular targets has deeply modified treatment strategies for patients with advanced NSCLC harboring mutations in epidermal growth factor receptor (EGFR), that predict both a better response to EGFR tyrosine-kinase inhibitors (TKIs: gefitinib, erlotinib, afatinib) and a longer progression-free survival which translates in increased overall survival. [5, 6, 7]. Nevertheless, body mass index, residual organ function and concomitant medications might affect either TKIs clearance in elderly patients or their efficacy and safety. However, clinical trials are conducted in selected patients aged 75 years or less that do not reflect people treated in clinical practice. Recently, several reports confirmed the safety and efficacy of gefitinib, a reversible EGFR tyrosine-kinase inhibitor, in mutated patients aged > 70 years, [8, 9] whereas no study has specifically evaluated afatinib, a selective irreversible ErbB family blocker, in this subset of patients. Pharmacokinetics characteristics of gefitinib and afatinib are different both in terms of bioavailability and drug metabolism that is influenced by individual variability. Their oral absorption is slow to moderate with substantial inter-individual differences in the extent of gastrointestinal tract mucosa. Gefitinib undergoes extensive hepatic metabolism predominantly by cytochrome P450 (CYP)-dependent enzymes; in contrast, afatinib undergoes minimal biotransformation and oxidative CYP-mediated metabolism is of negligible importance. Thus, gefitinib has an important potential for interaction with other agents that are metabolised by or are inhibitors/inducers of the CYP-related enzymes. Both gefitinib and afatinib undergo prevalent faecal excretion while renal elimination is responsible for < 5% of the administered dose. [10]

In the present retrospective analysis we have evaluated efficacy and safety of gefitinib and afatinib in elderly (≥ 70 years) compared to younger patients.

## RESULTS

### Patients' characteristics

Forty-nine out of 54 patients with histologically proven diagnosis of NSCLC (adenocarcinoma or other non-small cell histology) and with an EGFR gene mutation treated in our center between June 2010 and July 2014 were considered eligible. Most patients were female (74%) and never smokers (75%). Median age at diagnosis was 63 years (range 44-69) and 75 years (range 72-81) in group A and B, respectively; 8 out of 24 patients in group B (33%) had 80 years. Most of patients had a PS (ECOG) 1 (52% in group A and 54% in group B); performance status was equal or superior to 2 in 8% of patients in group A and in 25% of elderly patients. In group A, 20% of patients had no comorbidities, 72% had one or two comorbidities, 8% more than two. On the other hand, 67% of elderly patients had one or two comorbidities, 33% more than two comorbidities, 0% had no comorbidities. The most frequent concomitant diseases in the whole population were hypertension, diabetes and cardiac comorbidities (ischemic or arrhythmic cardiac disease); 6 patients of elderly group and 1 in the youngest group had a history of other malignancies within 5 years prior to diagnosis of NSCLC. In 90% of cases the histologic subtype of NSCLC was adenocarcinoma, in 10% adeno-squamous carcinoma. A total of 29 patients (59%) had a deletion in exon 19 (17 in group A and 12 in group B) and 20 (41%) had a point mutation in exon 21 (8 in group A and 12 in elderly group). All patients received a treatment with TKIs as first-line therapy. Out of 49 patients, 5 were treated with afatinib 40 mg/daily (1 in group A, 4 in group B) while 44 of them received gefitinib 250 mg/daily (24 in group A, 20 in group B). Treatment was continued until disease progression, unacceptable toxicity or patient's withdrawal. Patients' characteristics are summarized in Table 1.

**Table 1:** Patients' characteristics

	GROUP A (<70 years) *n*. 25	GROUP B (≥70 years) *n*. 24
***Median Age***	63 ( range 44-69)	75 (range 72-81)
***Smokers***	27%	25%
***Gender*** - Female	80%	76%
***PS (ECOG) at diagnosis***		
0	10/25 (40%)	5/24 (20%)
1	13/25 (52%)	13/24 (54%)
≥2	2/25 (8%)	6/24 (25%)
***Functional Impairment***		
- no comorbidities	5/25 (20%)	0/24 (0%)
- 1 or 2 comorbidities	18/25 (72%)	16/24 (67%)
- >2 comorbidities	2/25 (8%)	8/24 (33%)
***Most frequent comorbidities***		
Hypertension	12/25 (48%)	20/24 (83%)
Diabetes	4/25 (16%)	8/24 (33%)
Cardiac comorbidities	3/25 (12%)	4/24 (17%)
BPH*	1/25 (4%)	4/24 (17%)
History of other malignancies	1/25 (4%)	6/24 (25%)
Other	7/25 (28%)	8/24 (33%)
***First line treatment***		
Gefitinib	24/25 (96%)	20/24 (83%)
Afatinib	1/25 (4%)	4/24 (17%)
***EGFR mutation***		
19	17/25 (68%)	12/24 (50%)
21	8/25 (32%)	12/24 (50%)

* Benign prostatic hyperplasia

### Response and survival

**Figure 1:**
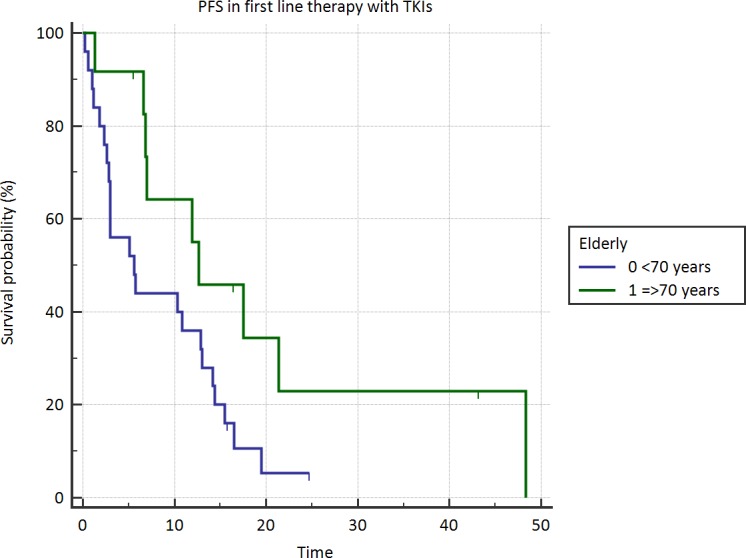
Progression free survival in elderly *vs* younger patients.

After a median follow up of 36 months, 23 patients in group A (92%) and 18 in group B (75%) had experienced disease progression after first-line TKI treatment and overall 7 are still alive (3 and 4 in group A and B, respectively). A significant difference in terms of median PFS between younger and elderly population was found: 5.6 and 12.6 months, respectively (*p* = .008; HR 0.46; 95% CI 0.24-0.87) (Figure 1). Conversely, OS was not significantly different between younger and elderly group (15.03 *vs*. 18.6 months; HR 0.76; 95%CI 0.42-1.38; *p* = .34). RR in group A was 64% in group A and 75% in group B; the difference was not significant (*p* = .89). No patient achieved a complete response. In group A 7/25 patients (28%) received a second-line treatment and 5/25 (20%) a third-line therapy, while only 4/24 (16.6%) and 2/24 (8.3%) of elderly patients received a second and third-line treatment, respectively.

The incidence of exon 21 point mutation, which is considered a negative prognostic factor in terms of survival in comparison to exon 19 deletions [11, 12, 13], was greater in elderly population than in group A (50% *vs* 32%). Despite this unfavourable distribution, in our analysis no difference in terms of PFS was found in elderly population carrying exon 19 and exon 21 mutations (14.3 *vs* 12.6 months, respectively; HR 0.82; 95%CI 0.32-2.12; *p* = .63). However, OS showed a trend toward an advantage in elderly patients with exon 19 deletions (22.4 months) in comparison to those carrying exon 21 point mutation (15.1 months); in spite of the apparently large difference, the result was not statistically significant (HR 0.79; 95%CI 0.34-1.82; *p* = .53).

An interesting finding was the difference in terms of median PFS of elderly patients receiving gefitinib compared to those receiving afatinib, which resulted significantly longer in the first ones (*p* < .0001). After a median follow up of 36 months, all patients (100%) treated with afatinib and 14 (70%) who received gefitinib experienced disease progression after first-line. Neverthess, this result is just a rough indication due to the small sample size of afatinib group.

### QoL and tolerability

Most patients had an improvement of performance status after treatment with TKIs, independently on age: 13 out of 25 in group A (52%) and 14 out of 24 elderly patients (58%). Eighteen (90%) out of 20 elderly patients treated with gefitinib experienced symptoms relief and upgrading of PS (ECOG) from a median of 2 to a median of 1.

The most frequent adverse events were rash, diarrhea and fatigue. There was no treatment-related death both in younger and older population and only few adverse events of grade 3/4, comparable in the two groups. There was a trend toward a more frequent and severe cutaneous rash between elderly patients (50% of all grades) than in younger population (40% of all grades), but the difference is not statistically significant. However, five patients of group B required dose reduction for treatment-related rash, six patients had a dose delay for diarrhea and one patient experienced interstitial lung disease, but there was no treatment interruption due to serious adverse event.

**Figure 2:**
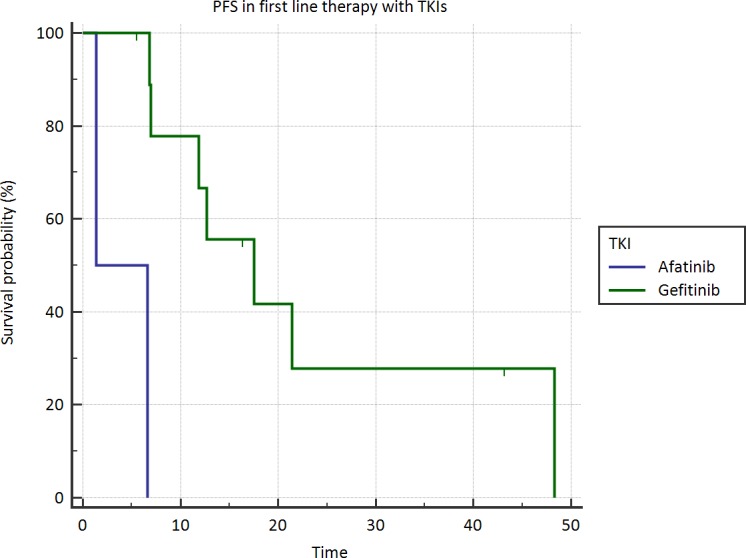
Progression free survival in elderly patients treated with gefitinib *vs* afatinib

## DISCUSSION

In the present study, treatment with TKIs seems more effective in elderly than in young patients affected by advanced NSCLC with an EGFR gene mutation; PFS was significantly longer in elderly, but OS was comparable in the two groups, suggesting that treatment with TKIs influences tumor response and prolongs the duration of first-line therapy but does not affect long-term survival, probably as a consequence of second and third line treatment, that were more common in the youngest population. No differences in terms of PFS were found in exon 21 *versus* exon 19 mutated elderly patients, but those carrying exon 19 deletions had an advantage in terms of OS (+7.3 months). Therefore, the known negative prognostic impact of exon 21 point mutations, [12, 13, 14] that were more frequent in elderly group, might have contributed to nullify the advantage in PFS. In a previous retrospective analysis, Wheatley-Price et al. demonstrated the efficacy of erlotinib in second or third-line comparing elderly and younger patients enrolled in BR.21 study; also in this case, it was found a better response rate (70% *vs* 59%) and a longer PFS (39 *vs* 34 weeks) in elderly group, but the difference was not statistically significant. [14]. Similarly, in POLARSTAR study elderly Japanese patients with previously treated NSCLC, not selected for EGFR mutations, showed a trend toward a longer PFS in comparison to younger group, even if not statistically significant.[15].

The reason for longer PFS in elderly patients is matter of discussion. Response to TKIs is correlated with EGFR mutations and EGFR gene copy number; there is no clear evidence of different EGFR expression in elderly even if in the analysis from Wheatly-Price a higher number of EGFR copies was found in elderly patients. However, this finding does not explain the longer PFS in elderly patients not selected for EGFR mutations and treated with TKIs in the POLARSTAR study.[15] Alternatively, body mass index, residual organ functions and metabolic effects of concomitant medications might affect TKIs clearance as well their efficacy and safety. No age-dependent effect has been reported on pharmacokinetic either of gefitinib or afatinib, [10] but it has been demonstrated that, in contrast to afatinib, gefitinib undergoes extensive hepatic metabolism by cytochrome P450 (CYP)-dependent enzymes suggesting the possibility of significant interactions with other agents metabolised by CYP-related enzymes. By the contrary, mild and moderate hepatic impairment, as it might be observed in patients with liver metastases, does not seem to have a major impact on exposure of TKIs. Moreover, both gefitinib and afatinib have a prevalent faecal excretion with < 5% of drug excreted in the urine, so excluding that a mild or moderate renal impairment might influence the exposure to TKIs. Therefore, excluding effects of age- or cancer-related impairment of renal and hepatic function on a greater exposure to afatinib and gefitinib in elderly patients, we hypothesize that polypharmacy might be responsible of an altered clearance of TKIs. Drugs interacting both with transporter molecules at enterocyte level as P-gp (ATP-binding cassette drug transporter P-glycoprotein1) and liver metabolizing CYP-dependent enzymes affects bioavailability of TKIs. Gefitinib and afatinib are a substrate and afatinib is also an inhibitor of P-gp; strong P-gp inhibitors (ketoconazole, itraconazole, amiodarone, verapamil etc) can increase exposure to afatinib, especially if administered nearby the TKIs. However, most interactions involve drug-metabolizing CYP-related enzymes and gefitinib; CYP-inhibitors (ciprofloxacin, clarithromycin, dexamethasone, fluconazole, metronidazole, ketoconazole, omeprazole, ranitidine, valproic acid etc) could increase plasma levels of gefitinib, thus inducting both a better therapeutic activity and a greater percentage of adverse reactions. Moreover the activity of these enzymes could be constitutively reduced in elderly by age. [16, 17]. Retrospective studies suggest that the severity of rash or diarrhea correlates with exposure to TKIs. [18, 19]. Actually, in our study elderly patients experienced more toxicities and were more likely to have grade ≥ 3 rash, fatigue and diarrhea, suggesting a higher plasma concentration of TKIs in this group. Moreover, our data suggest a trend toward a longer PFS in elderly patients who underwent gefitinib rather than afatinib - which is not an inhibitor or inducer of CYP-related enzymes - probably due to the different metabolism and drug interactions, although these results are biased by the small sample size of the afatinib subgroup. Since it was demonstrated in phase I studies that gefitinib 250mg/die is about one-third of the maximum tolerated dose, [20]. we can assume that gefitinib might be more effective than afatinib in elderly because concomitant medications cause a higher but tolerable plasma concentration of this TKI.

In conclusion the present study shows that first-line therapy with TKIs is feasible in elderly population and is more effective in terms of response rate and PFS in comparison to the younger subgroup. The combined effect of concomitant medications on transport and metabolism of gefitinib and afatinib may induce increased plasma levels of TKIs, that translate in greater efficacy and toxicity.

## PATIENTS AND METHODS

### Patients' selection

This retrospective analysis includes 49 patients (aged ≥18 years) with histologically proven metastatic NSCLC and mutations in exon 19 or 21 treated in our center between 2010 and 2014. All mutational analyses were conducted in the laboratory of Diagnostic Molecular Pathology at the Catholic University of Sacred Heart (Rome, Italy). Taking into account the age at the beginning of therapy, patients were divided into two groups: group A (patients aged < 70 years) and group B (patients aged ≥ 70 years). Inclusion criteria for selection were: a) first-line therapy with TKIs (gefitinib or afatinib); b) imaging assessment (Computed Tomography or Positron Emission Tomography-Computed Tomography) performed at regular intervals (no longer than 3 months); c) complete informations regarding comorbidities and concomitant medications. Data on patients' functional status (performance status - PS ECOG), comorbidities and concomitant medications have to be available at the time of analysis. Patients were excluded if harboring more than one mutation in two or more exons or in case of resistance mutations (p.T790M, exon 20 insertion). Patients whose diagnosis was performed after November 2014 were excluded in order to assure a minimum follow-up of at least one year. Finally, patients treated within clinical trials with any drug not previously approved were not included in the analysis. The study has been conducted in accordance with the rules of the local Ethics Committee and the Declaration of Helsinki. All patients provided a written consent for use of their clinical data; a separate consent for molecular analyses was obtained.

### Immunohistochemistry and DNA mutation analysis

Formalin-fixed paraffin-embedded samples were obtained before starting any cancer therapy as a set of ten 5-μm slides or as uncut tissue blocks. Mutational analysis was performed by Sanger sequencing or by Therascreen EGFR RGQ PCR Kit (Qiagen, Hilden, Germany). Genomic DNA was extracted from tumors lung tissue using the QIAamp DNA FFPE Tissue Kit (Qiagen) according to the manufacturer's protocol. For Sanger sequencing EGFR genes (exons 18, 19, 20 and 21) were amplified using the following primers: for exon 18, forward 5′-TCC AAA TGA GCT GGC AAG TG-3′ and reverse 5′-TCC CAA ACA CTC AGT GAA ACA AA-3′; for exon 19, forward 5′-GTG CAT CGC TGG TAA CAT CC-3′ and reverse 5′-TGT GGA GAT GAG CAG GGT CT-3′; for exon 20, forward 5′- ATC GCA TTC ATG CGT CTT CA-3′ and reverse 5′- ATC CCC ATG GCA AAC TCT TG-3′; for exon 21, forward 5′- GCT CAG AGC CTG GCA TGA A-3′ and reverse 5′- CAT CCT CCC CTG CAT GTG T-3′. PCR conditions were as follows: initial denaturation at 95°C for 10 min followed by 35 cycles at 95°C for 40 sec, 50°C for 40 sec and 72°C for 40 sec. After visualization onto agarose gel, PCR products were treated with ExoSAP-IT (USB Corp., Cleveland, OH, USA) following the manufacturer's protocol, amplified with BigDye Terminator version 3.1 cycle sequencing kit (Applied Biosystems) using forward and reverse primers and sequenced with an ABI PRISM 3100-Avant Genetic Analyzer (Applied Biosystems). For the therascreen EGFR RGQ PCR Kit (Qiagen) 10 ng of DNA was amplified by real-time PCR in 25 μL reactions according to the manufacturer's protocol. Real-time PCR was performed using the Rotor-Gene Q 5plex HRM (QIAGEN). The cycling conditions were as follows: 95°C 10 min and 40 cycles at 95°C 30 s, 60°C 60s. The software program Rotor-Gene Q 2.0.2 was used to process the data. The sample Ct was compared with the cut-off point for the specific assay (cut-off ΔCt) according to instructions in the manual.

### Statistical analyses

Primary endpoints were progression free survival (PFS), response rate (RR) and clinical benefit in terms of quality of life (QoL) after first-line therapy with TKIs; overall survival (OS) was considered as secondary endpoint. PFS was calculated from the beginning of first-line therapy until radiologically assessed disease progression or treatment discontinuation for unacceptable toxicity or patient's withdrawal. OS was measured from diagnosis of metastatic disease until death or last follow-up contact. The outcome was censored if a patient had not progressed or was not dead at the time of last follow-up. Clinical response to treatment was classified as complete response, partial response, stable disease or progressive disease according to the Response Evaluation CriteriaIn Solid Tumors (RECIST) version 1.1.[21]. Kaplan-Meier method and the log-rank test were used to estimate PFS and OS. All reported p values are two-tailed and a level of 0.05 or less was considered statistically significant.

## SUPPLEMENTARY MATERIAL


