# Supplementary material for: Are TKIs favourable for the elderly with non-small-cell lung cancer?
Source: Oncotarget. 2016 May 17;7(30):46871–7. doi: 10.18632/oncotarget.9389 (PMC5216909; doi:10.18632/oncotarget.9389)
Supplement: Supplementary file 1 [file oncotarget-07-46871-s001.pdf]

Are tkis favourable for the elderly with non-small-cell lung cancer?

Supplementary Material

Another possible hypothesis regards the potential benefit of inhibition of some intracellular signaling pathways in elderly population. Recent studies have revealed that IGF-1, RAS, MEK, PI3K, mTOR and other signaling molecules targeted by TKIs are involved in the aging process and in gerosuppression. In fact, aging is not driven by DNA damage but instead is driven by sensing-signaling pathways governing cellular metabolism and growth and these signaling pathways are identical to oncogenic pathways that drive cancer.[1] [2]

EGFR stimulates intracellular signaling cascades, such as the RAS/RAF/ERK (MAPK) pathway and the PI3K/AKT/mTOR pathway. The PI3K/mTOR pathway is almost universally activated in cancer; when the cell cycle is blocked, yet mTOR is active, cells undergo gerogenic conversion or geroconversion, that is a transition from cell cycle arrest and quiescence to senescence.[3] Analogs of rapamycin as well as EGFR inhibitors block the signal transduction through PI3K/mTOR pathway and suppress geroconversion. *In vitro* studies demonstrated that an increased expression of mTOR predicted a lower sensitivity to gefitinib; thus, hyperactivity of mTOR pathway is one of the mechanisms of acquired resistance to gefitinib [4]

Inhibition of the mTOR pathway extends lifespan in all species studied to date, and in mice delays the onset of aging-related diseases and co-morbidities.[5] [6] In a recent study has been shown that rapamycin analogs ameliorated immunosenescence (i.e. the decline in immune function during aging) in elderly volunteers, as assessed by their response to influenza vaccination. In fact rapamycin analogs enhanced the response to the influenza vaccine by about 20%, reducing the percentage of CD4 and CD8 T lymphocytes expressing the programmed death-1 (PD-1) receptor, which inhibits T cell signaling and is more highly expressed with age. These results raise the possibility that mTOR inhibition may have beneficial effects on immunosenescence particularly in the elderly. [7]

---

[1] Blagosklonny MV. Rapamycin extends life- and health span because it slows aging. Aging (Albany NY). 2013;5(8):592-8.

[2] Blagosklonny MV. Selective anti-cancer agents as anti-aging drugs. Cancer Biol Ther. 2013;14(12):1092-7.

[3] Blagosklonny MV. Rapalogs in cancer prevention: anti-aging or anticancer? Cancer Biol Ther. 2012;13(14):1349-54.

[4] Karachaliou N, Codony-Servat J, Teixidó C et al. BIM and mTOR expression levels predict outcome to erlotinib in EGFR-mutant non-small-cell lung cancer. Sci Rep. 2015;5:17499.

[5] Popovich IG, Anisimov VN, Zabezhinski MA, Semenchenko AV, Tyndyk ML, Yurova MN , Blagosklonny MV. Lifespan extension and cancer prevention in HER-2/neu transgenic mice treated with low intermittent doses of rapamycin. Cancer Biol Ther. 2014;15(5):586-92.

[6] Mercier I, Camacho J, Titchen K et al. Caveolin-1 and accelerated host aging in the breast tumor microenvironment: chemoprevention with rapamycin, an mTOR inhibitor and anti-aging drug. Am J Pathol. 2012;181(1):278-93.

[7] Mannick JB, Del Giudice G, Lattanzi M et al. mTOR inhibition improves immune function in the elderly. Sci Transl Med. 2014;6(268):268ra179.
